# Different resting-state network disruptions in newly diagnosed drug-naïve Parkinson’s disease patients with mild cognitive impairment

**DOI:** 10.1186/s12883-021-02360-z

**Published:** 2021-08-25

**Authors:** Yanbing Hou, Qianqian Wei, Ruwei Ou, Lingyu Zhang, Xiaoqin Yuan, Qiyong Gong, Huifang Shang

**Affiliations:** 1 Department of neurology, Laboratory of Neurodegenerative Disorders, National Clinical Research Center for Geriatrics, West China Hospital, Sichuan University, Chengdu, Sichuan China; 2 Department of Neurology, Mianyang Central Hospital, School of Medicine, University of Electronic Science and Technology of China, Chengdu, 610041 Sichuan China; 3 Huaxi MR Research Center (HMRRC), Department of Radiology, West China Hospital, Sichuan University, Chengdu, Sichuan China

**Keywords:** Parkinson’s disease, Resting state functional MRI, Independent component analysis, Functional connectivity, Mild cognitive impairment

## Abstract

**Background:**

Cognitive impairment is a common non-motor symptom in patients with Parkinson’s disease (PD). Mild cognitive impairment (MCI) is also prevalent in nondemented PD patients, even in newly diagnosed PD patients. The possible impacts of MCI on brain function activities for PD patients need more investigation, and the potential of emerging technologies for detecting underlying pathophysiology of cognitive signs in PD can be further improved.

**Method:**

Forty-seven newly diagnosed drug-naïve PD patients (28 PD-MCI patients and 19 PD patients with cognitively unimpaired (PD-CU)) and 28 healthy controls (HCs) underwent resting-state functional MRI. The connectivity patterns of specific networks were investigated through the independent component analysis among PD-MCI, PD-CU and HCs groups.

**Results:**

The independent component analysis revealed significantly decreased functional connectivity (FC) of the default mode network, visual network and sensorimotor network in the PD-MCI subgroup compared with the HC group. Furthermore, FC of the default mode network was positively correlated with memory scores from the brief visuospatial memory test-revised, and FC of the visual network was positively correlated with visuospatial scores from the clock copying test in the PD-MCI group. In all patients with PD, FC of the sensorimotor network negatively correlated with motor severity scores from the Unified PD Rating Scale (UPDRS) part III. On the other hand, the potential damage was more likely to occur in FC between the sensorimotor network and limbic network, and between the ventral attention network and visual network in all PD patients.

**Conclusions:**

Newly diagnosed drug-naïve PD-MCI patients showed characteristic damage of FC within the default mode network, visual network and sensorimotor network, and all PD patients presented impaired FC between the sensorimotor network and limbic network, and FC between the ventral attention network and visual network. These network-wide functional aberrations may underline the pathophysiology of PD.

**Supplementary Information:**

The online version contains supplementary material available at 10.1186/s12883-021-02360-z.

## Introduction

Parkinson’s disease (PD), as the second most common neurodegenerative disorder, is characterized by motor symptoms and a broad spectrum of non-motor symptoms (NMS). Cognitive impairment has been one of the most frequent non-motor features of PD. Dementia has high prevalence (up to 80%) in PD patients with long disease course [1, 2], and mild cognitive impairment (MCI) is also prevalent in nondemented PD patients with a mean of 26.7% (range 18.9–38.2%) [3]. Another study reported that the frequency of MCI even exceeded 40% in a large cohort of newly diagnosed PD patients [4]. PD with MCI is at a higher risk of progression to PD with dementia (PDD) that can contribute to a poorer quality of life [5] and has attracted lots of attention in recent decades.

The formation of protein aggregates (including the deposition of neurofibrillary tangles, tau and amyloid-β), dysfunction of neurotransmitter systems and genetic mutations (e.g., *MAPT*, *GBA*) are closely related to cognitive dysfunction in PD [6]. In addition, there is a“dual syndrome hypothesis”, that is, dopaminergic dysfunction in fronto-striatal regions and cholinergic dysfunction in temporal and posterior cortical regions. Specifically, the fronto-striatal dysfunction is related to deficits in planning, working memory and executive function, and the temporal and posterior cortical dysfunction is associated with deficits in visuo-spatial function and semantic fluency [7]. Although the underlying pathology of cognitive dysfunction in PD has been widely studied, no effective biomarker for predicting PD-MCI or PDD has been confirmed. Currently, low-level epidermal and insulin-like growth factors or uric acid in plasma/serum, low-level Aß in CSF, decreased cerebral cholinergic innervation or metabolism mainly in posterior regions of brain evaluated by positron emission tomography (PET), and atrophic hippocampus assessed by magnetic resonance imaging (MRI) are significant markers to indicate the risk of dementia in PD patients [8].

A number of neuroimaging techniques have been employed to explore pathological substrates of PD and other neurodegenerative disorders [6]. The resting-state functional MRI (rs-fMRI), one promising technology with reliability and reproducibility, can assess regional blood-oxygen-level-dependent (BOLD) signal fluctuations and reflect the baseline functional cerebral architecture [9]. Seed-based connectivity methods can evaluate the functional connectivity (FC) of different brain regions relying on a certain hypothesis. Our previous studies based on a priori seed-based analysis to characterize FC changes in the default mode network (DMN) in drug-naïve PD patients with MCI [10, 11]. On the other hand, data-driven approaches can identify coherent spatial patterns of BOLD signals without a specified model. Independent component analysis (ICA), one of the most frequently data-driven technique, can blindly separate underlying signals into independent components (ICs) and characterize the functional activities in cerebral resting state networks (RSNs) [12]. These RSNs have different brain function and display specific functional alterations for a particular disease [13]. As one of the famous RSNs, DMN is linked to self-referential or internally-directed cognitive states and has been reported to be associated with attention/working memory and memory performance [14]. Other RSNs, including the fronto-parietal network (FPN), dorsal attention network (DAN) and ventral attention network (VAN), are also relevant for cognitive processes [15, 16]. Various studies have investigated these RSNs in PD-MCI or PDD and described several alterations in functional network connectivity. Through the data collection from available studies, a meta-analysis including seventeen studies found that PD patients with cognitive impairment had a reduced connectivity mainly in the DMN [6].

However, most of previous studies involved patients who had been chronically taking anti-Parkinson medication and performed in the OFF or ON state. Long-term use of anti-Parkinson drugs may lead to reorganization of FC, which may not reflect primary pathophysiological changes [17]. Therefore, drug-naïve PD patients may be crucial to elucidate the disruption of RSNs in PD with MCI. In this current study, we planned to quantify changes in the intra and inter-network connectivity in newly diagnosed drug-naïve PD patients with and without MCI using the ICA. We supposed that PD-MCI patients would show disrupted FC in both within and between several cognitive-related networks in the newly diagnosed drug-naïve state. Furthermore, we aimed to assess relationships between these alterations in RSNs and cognitive performance (including executive, attention/working memory, memory, language and visuospatial functions).

## Materials and methods

### Participants

Our study was approved by West China Hospital of Sichuan University Clinical trials and Biomedical ethics committee with the reference number of 2013 (243), and we got the written informed consents from all participants. Patients were recruited consecutively in our cohort at the Movement Disorders Outpatient Clinic of West China Hospital of Sichuan University. Healthy controls (HCs) in our cohort were unrelated family members (e.g., spouse) of patients recruited through advertisements. Regretfully, we did not record and summarize in detail the reasons why patients or HCs refused to participate in the study, but we found that (1) Most patients agreed to participate in our study; (2) Some of unrelated family members of patients were not interested in the study; (3) Patients were more interested in the study than HCs. Finally, a total of 75 subjects were enrolled including 47 newly diagnosed drug-naïve PD patients and 28 HCs. All patients met the United Kingdom PD Society Brain Bank criteria for PD, and were reconfirmed after 1 to 2 year from the first assessment. PD patients were excluded if they had a history of other neurological or psychiatric disease, moderate or severe head tremor, and any disorders that can interfere with symptom assessment. HCs had normal neurological status and brain structure, absence of any history of neurological or psychiatric disorders.

Motor disease severity was assessed by the unified PD rating scale (UPDRS) [18] and the Hoehn & Yahr stage (H&Y) [19]. The NMS was evaluated by the NMS scale (9 domains) [20], the Hamilton depression rating scale (HDRS) [21], and the Hamilton anxiety rating scale (HARS) [22]. The Montreal cognitive assessment (MoCA) was used to assess the global cognitive function [23]. In addition, overall cognitive functions of all participants were assessed by the complete neuropsychological battery (5 domains) [24], (1) attention/working memory (adaptive digit ordering test (DOT-A) and backward digit span test (DST)); (2) executive function (verbal fluency test (VFT) and clock drawing test (CDT)); (3) language (Similarity test in Wechsler intelligence scale for adult-Chinese revised (WAIS-RC) and Boston naming test (BNT)); (4) memory (Hopkins verbal learning test-revised (HVLT-R) and brief visuospatial memory test-revised (BVMT-R)); and (5) visuospatial function (Benton line orientation (BLO) and clock copying test (CCT)) [24] (details were seen in the Supplementary material.1 (Suppl.1)). Based on the HC group’s means and standard deviations (SD), we calculated actual z-scores for PD patients (details were seen in the Suppl.2). Meantime, expected z-scores for PD patients were calculated with age, gender, and level of education adjusted in a multiple regression analysis. Patients were classified as having MCI if the actual z-score for a test was more than 1.5 standard lower than the expected score in at least two tests in one domain or in one test per domain in at least two domains [25, 26]. In the present study, 28 PD patients diagnosed as having MCI (the PD-MCI subgroup) and 19 PD patients diagnosed as cognitively unimpaired (the PD-CU subgroup).

### MRI data acquisition

We used a three Tesla system (Tim Trio; Siemens Healthineers, Erlangen, Germany), equipped with an eight-channels head coil, to collect functional and conventional MRI images. The resting-state fMRI data was acquired using an echo-planar-imaging (EPI) sequence. Repetition time [TR] = 2000 ms, echo time [TE] = 30 ms, flip-angle [FA] = 90°, field-of-view [FOV] = 240 × 240 mm^2^, matrix size = 64 × 64, voxel size = 3.75 × 3.75 × 5 mm^3^, axial slices = 30, number of time points = 240. The conventional MRI data including axial T1-weighted, T2-weighted and fluid-attenuated inversion recovery imaging was collected (details were seen in the Suppl.1). All subjects were instructed to lie comfortably on the scanner bed, and be awake with their eyes closed.

### FMRI data preprocessing

All fMRI volumes underwent a preprocessing based on SPM12 (http://www.fil.ion.ucl.ac.uk/spm) and DPABI 3.0 (http://www.rfmri.org/dpabi), including (1) removing the first 10 time points; (2) slice-timing correction; (3) spatial realignment; (4) spatial normalization into the standard Montreal Neurological Institute (MNI) space and resampling into 3 × 3 × 3 mm^3^; (5) spatial smoothing with a 6 mm full-width half-maximum (FWHM) isotropic Gaussian kernel. In addition, the displacement and angular rotation of all participants in the x, y, or z plane were < 1.0 mm and < 1.0° respectively.

### Ica

**Fig. 1:**
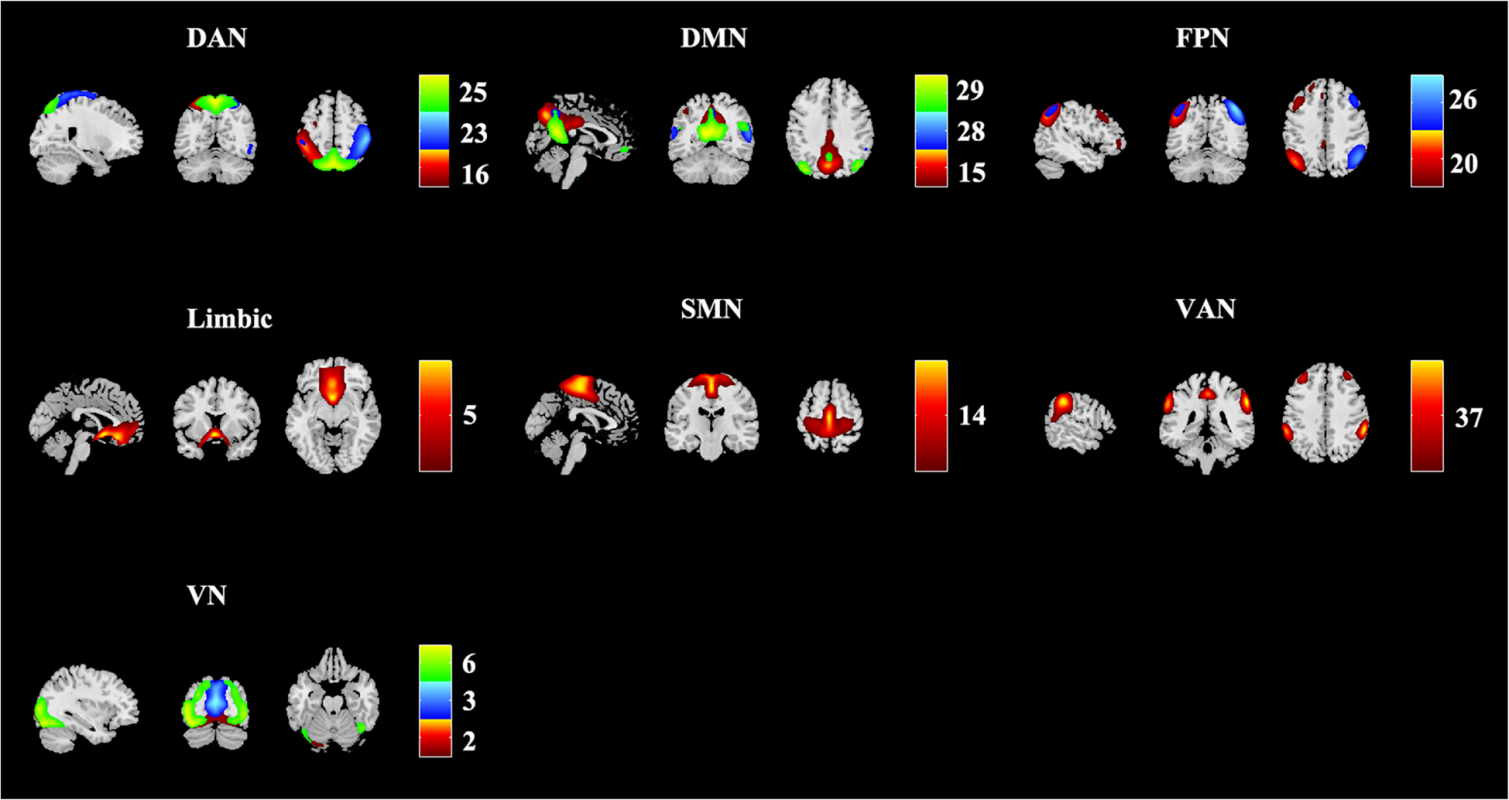
Resting state networks (RSNs) inferred using group ICA. Fourteen independent components (ICs) were anatomically labelled into 7 RSNs. Keys, DAN, dorsal attention network; DMN, default mode network; FPN, fronto-parietal network; SMN, sensorimotor network; VAN, ventral attention network; VN, visual network. Color bar means different numbered ICs

The group ICA (gICA) was used for all subjects, through the GIFT toolbox (v3.0a) (http://mialab.mrn.org/software/gift/) with several steps: (1) estimating the number of ICs by the MDL criterion [27]; (2) auto-filling data reduction values; (3) selecting a neural network algorithm (Infomax); (4) repeating 250 times in ICASSO [28]; (5) running gICA serially. GICA3 back-reconstruction was performed to estimate spatial maps and timecourses for each subject. The spectral characteristics of component timecourses and the quality index (Iq) were used to select reliable RSNs from the physiological noise. The spectral characteristics include two parameters (“dynamic range” and “low frequency to high frequency power ratio”). And Iq shows the reliability and consistency of the decomposition with range from 0 to 1. Then the component was excluded when it has at least one of the following metrics: (1) dynamic range < 0.025 (2) ratio < 3.4; (3) Iq < 0.9 [29]. Then, 14 remaining ICs were anatomically labeled according to the correlation sorting criteria with the spatial correlation between the spatial map of each IC and the template from a previous study containing 7 main brain functional networks (DAN, DMN, FPN, limbic network, sensorimotor network (SMN), VAN, and visual network (VN)) (see in the Fig. 1) [30].

### FC analysis

The FC analysis was based on SPM12 and DPABI 3.0 with steps after the fMRI data preprocessing: (1) linear detrending; (2) nine nuisance covariates (the white matter (WM) signal, cerebrospinal fluid (CSF) signal, global signal and six head motion parameters) regression; (3) bandpass filtering (0.01–0.08 Hz). Then, these 14 ICs divided into 7 RSNs were regarded as 7 binary masks to analyze the Pearson’s correlation coefficient between each pair of RSNs, and z-scores were computed via a Fisher r-to-z transformation.

### Statistical analysis

Demographic and clinical data were performed by the one-way analysis of variance (ANOVA), student’s t-test, nonparametric test, or Chi-square test, as appropriate through SPSS 22.0. For intra-connectivity within each RSN, we used a design model of one-way ANOVA base on SPM12 to compare z-value maps among PD-MCI, PD-CU and HC groups with age, gender and level of education as covariates, and then the post hoc two-sample t-tests were performed (*p* < 0.001 at the voxel level and *p* < 0.05 at the cluster level corrected by Gaussian Random Filed (GRF), one-tailed) (http://restfmri.net/forum/index.php?q=rest). In addition, voxel-wise correlation analysis with clinical and cognitive scores was assessed based on RESTplus 1.2 (http://restfmri.net/forum/index.php?q=rest) with age, sex and education as covariates (p < 0.05, uncorrected). For inter-connectivity between each RSN, we also used the ANOVA by SPSS 22.0 among three groups with post-hoc t tests.

## Results

The demographic and clinical data of all participants were listed in Table 1. There was no significant difference in age, gender and level of education between PD patients and HCs. No significant difference was found in disease duration, H&Y stage, UPDRS score, NMS scores and HDRS/HARS scores between PD-MCI and PD-CU subgroups. Notably, the level of education was lower in the PD-MCI subgroup relative to the PD-CU subgroup.

**Table 1:** Demographic and clinical characteristics of all subjects

Parameter	PD, all	HC	PD-CU	PD-MCI	P^1^	P^2^	P^3^
Number, n	47	28	19	28	–	–	–
Handedness of writing (R: L)	47: 0	28: 0	19: 0	28:0	–	–	–
Age, y	55.00 ± 9.27	52.06 ± 7.00	54.88 ± 10.89	55.07 ± 8.20	0.085^#^	0.361	0.947^#^
Gender, M/F	23/24	8/20	8/11	15/13	0.083	0.164	0.440
Duration of disease, years	1.74 ± 1.61	–	1.87 ± 1.32	1.65 ± 1.80	–	–	0.288^#^
H & Y stage	–	–	1.87 ± 0.37	1.82 ± 0.58	–	–	1.000^#^
UPDRS score	–	–	–	–	–	–	–
Part I	–	–	0.79 ± 1.72	0.86 ± 1.21	–	–	0.448^#^
Part II	–	–	6.63 ± 3.18	6.21 ± 4.09	–	–	0.710
Part III	–	–	20.79 ± 7.44	19.79 ± 8.71	–	–	0.683
Part IV	–	–	0	0	–	–	–
NMSS score			21.95 ± 20.82	25.96 ± 21.73			0.380^#^
HDRS score	–	–	5.21 ± 6.87	6.79 ± 5.32	–	–	0.112^#^
HARS score	–	–	3.79 ± 4.88	5.68 ± 4.30	–	–	0.058^#^
MoCA score	–	–	27.89 ± 2.49	24.89 ± 3.28	–	–	0.002*
EDU, years	10.04 ± 4.01	10.29 ± 3.75	12.53 ± 3.03	8.36 ± 3.74	0.757^#^	0.001*	< 0.001*^#^

*Indicates significant difference;

^#^ Indicates the nonparametric test because of the non-normal distribution;

^1^ Comparison between all PD patients (PD-MCI and PD-CU) and HCs

^2^ Comparison among PD-MCI, PD-CU patients, and HCs

^3^ Comparison between PD-CU and PD-MCI patients

Keys: *PD* Parkinson’s disease; *HC* healthy control; *PD-CU* PD patients with cognitive unimpaired; *PD-MCI* PD patients with mild cognitive impaired; *R* right; *L* left; *M* male; *F* female; *H & Y* Hoehn & Yahr; *UPDRS* unified PD rating scale; *NMS* non-motor symptoms; *HDRS* Hamilton depression rating scale; *HARS* Hamilton anxiety rating scale; *MoCA* Montreal cognitive assessment; *EDU* education

The DAN was characterized by IC 16, IC 23 and IC 25 in bilateral superior parietal lobules; the DMN was labelled with IC 15, IC 28 and IC 29 in bilateral post cingulate cortices and precuneus; the FPN was marked by IC 20 and IC 26 in bilateral inferior parietal lobules; the limbic network was comprised of IC 5 in bilateral rectus, olfactory cortices and orbitofrontal gyri; the SMN was composed of IC 14 in bilateral postcentral gyri, precentral gyri, paracentral gyri and supplementary motor areas (SMA); the VAN was presented by IC 37 in bilateral middle cingulate cortices; and the VN was showed by IC 2, IC 3 and IC 6 in bilateral calcarine and lingual areas (see in the Fig. 1).

**Fig. 2:**
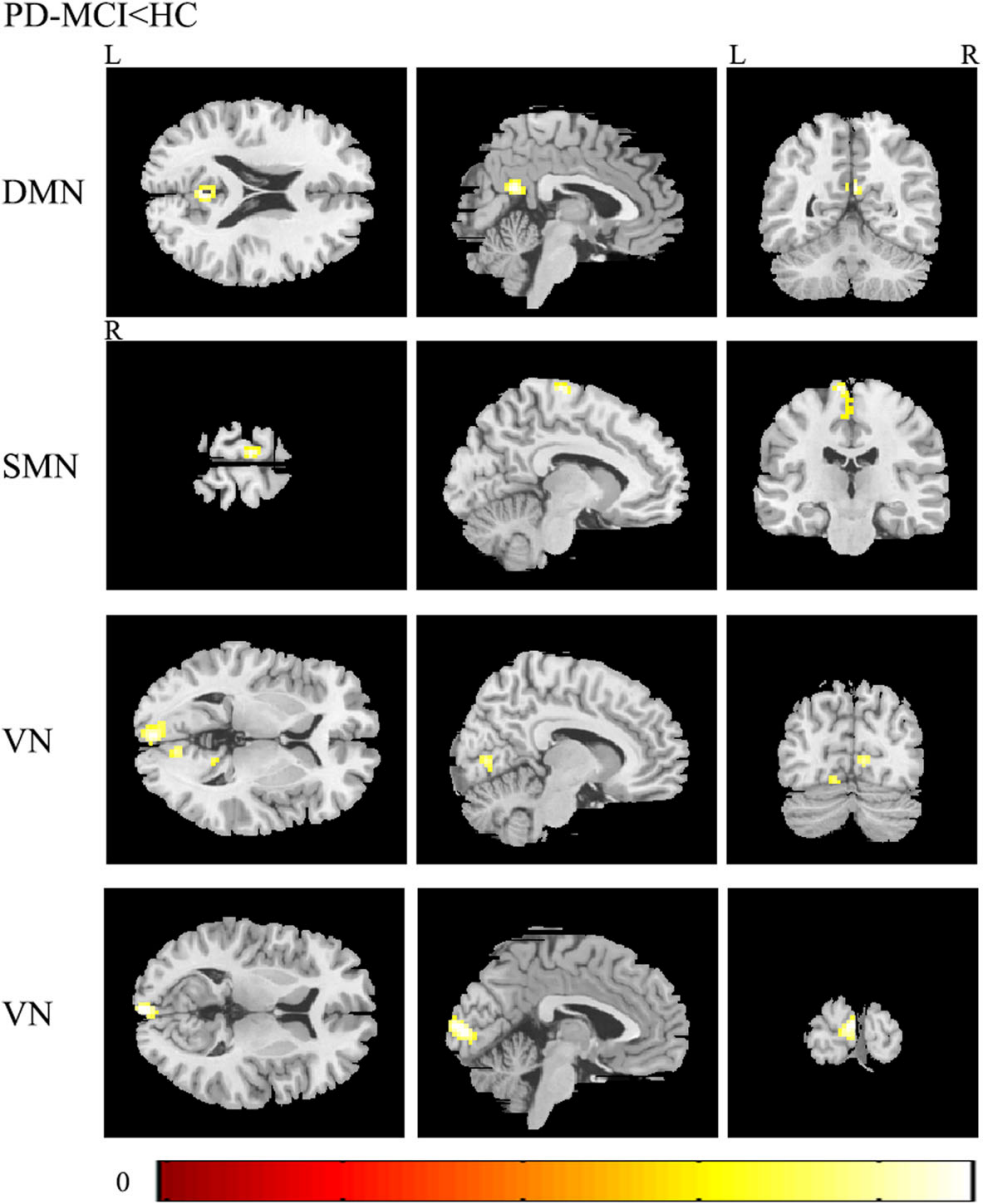
Differences in the connectivity patterns are shown between PD-MCI patients and HCs within the DMN, SMN and VN (*p* < 0.001, Gaussian Random Filed (GRF) corrected). Keys, DMN, default mode network; HC, healthy control; L, left; MCI, mild cognitive impairment; PD, Parkinson’s disease; R, right; SMN, sensorimotor network; VN, visual network

**Fig. 3:**
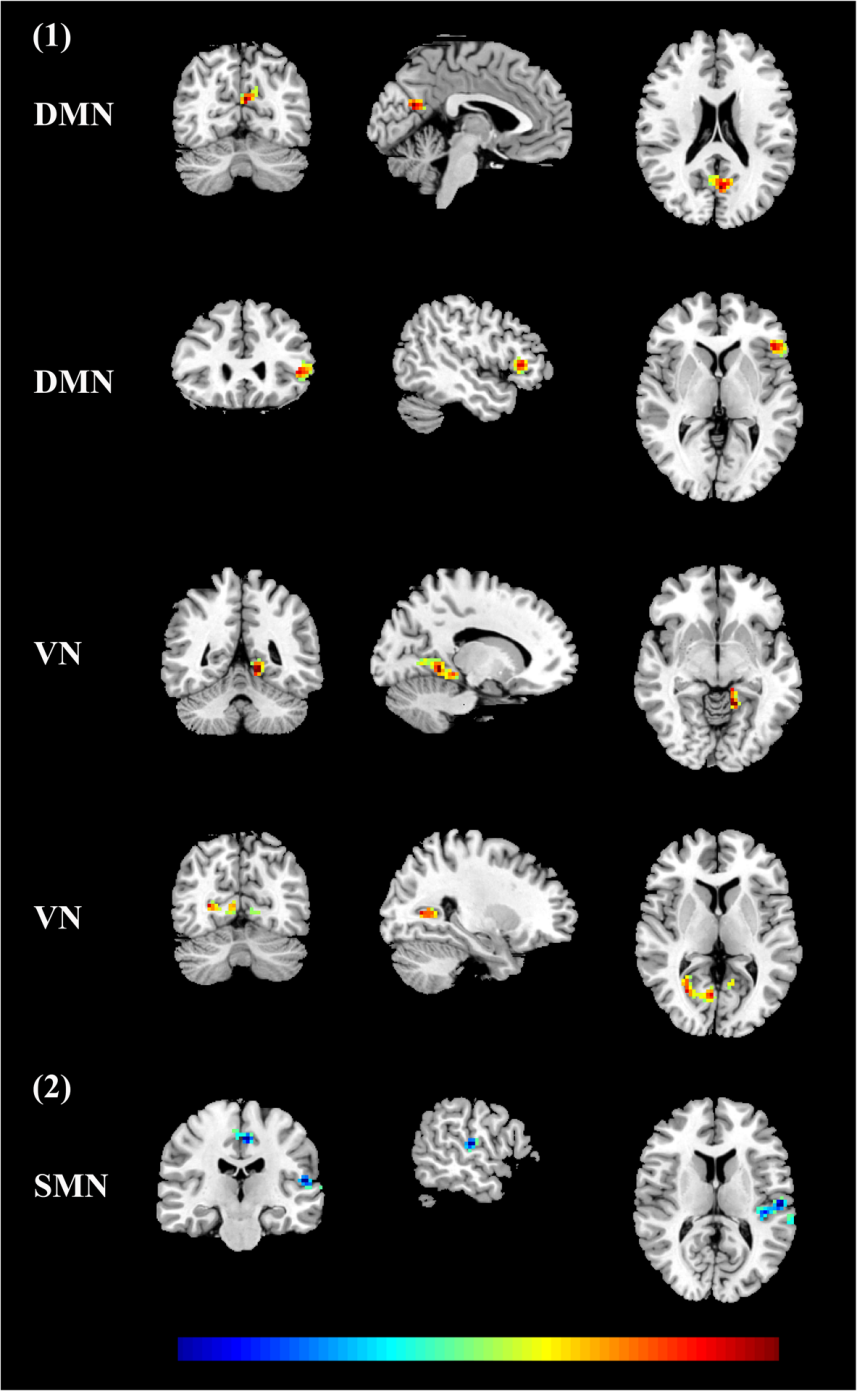
(1) In PD-MCI patients, FC within the DMN was positively correlated with BVMT-R scores, and FC within the VN was positively correlated with CCT scores; (2) In all PD patients, FC within the SMN was negatively correlated with UPDRS part III scores. Keys, BVMT-R, brief visuospatial memory test-revised; CCT, clock copying test; DMN, default mode network; FC, functional connectivity; MCI, mild cognitive impairment; PD, Parkinson’s disease; SMN, sensorimotor network; UPDRS, the unified Parkinson’s disease rating scale; VN, visual network

**Fig. 4:**
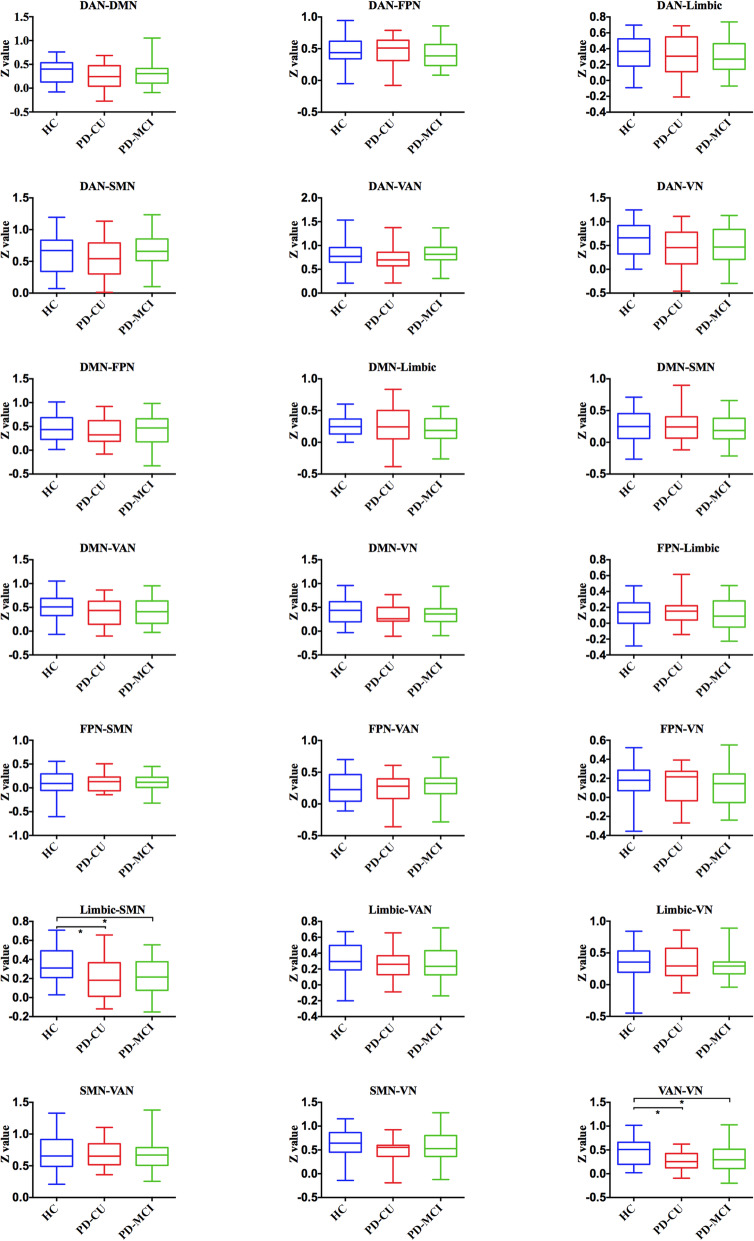
Significantly altered FC among PD-MCI, PD-CU and HC groups was indicated by an asterisk (*p < 0.05). Keys, DAN, dorsal attention network; DMN, default mode network; FPN, fronto-parietal network; HC, healthy control; MCI, mild cognitive impairment; PD, Parkinson’s disease; SMN, sensorimotor network; VAN, ventral attention network; VN, visual network

We found abnormal intra-network connectivity in PD-MCI patients. Compared with HCs, PD-MCI patients displayed lower intrinsic activities in the right precuneus of the DMN, left paracentral gyri and SMA of the SMN, and bilateral calcarine and lingual areas of the VN (see in the Fig. 2 and Table 2). Meantime, the BVMT-R scores (memory) were positively correlated with intrinsic activities in the precuneus within the DMN in PD-MCI patients; the CCT scores (visuospatial performance) were positively correlated with intrinsic activities in the calcarine and lingual areas within the VN in PD-MCI patients (see in the Fig. 3 (1) and Table 3). For all PD patients, the UPDRS part III scores were negatively correlated with intrinsic activities in areas of the SMN (see in the Fig. 3 (2) and Table 3). On the other hand, we detected abnormal FC between any two RSNs. Specifically, compared with the HC group, both PD-MCI and PD-CU subgroups had lower FC between the SMN and limbic network, and between the VAN and VN (see in the Fig. 4).

**Table 2:** Clusters derived from voxel-based comparisons between PD-MCI patients and HCs

RSNs	N	Size	Region	T		MNI	
					X	Y	Z
DMN	1	36	Precuneus_R	4.69	3	-54	21
SMN	1	65	Paracentral lobule_L	5.19	−9	−21	75
			Supplementary motor area_L	3.96	−3	−18	60
VN	1	122	Calcarine_L	4.51	−3	−96	9
			Calcarine_L	4.06	−9	−84	−3
			Lingual_L	4.03	−15	−69	−9
	2	62	Lingual_R	4.04	9	−75	3
			Lingual_R	3.88	15	−66	−3
			Lingual_R	3.66	21	−66	−9

Keys, *DMN* default mode network; *HC* healthy control; *L* left; *MCI* mild cognitive impairment; *PD* Parkinson’s disease; *R* right; *SMN* sensorimotor network; *VN* visual network

**Table 3:** Clusters derived from voxel-wise correlation analyses

RSN	N	Size	Region	T value		MNI	
					X	Y	Z
BVMT-R scores							
DMN	1	68	Precuneus	0.71	−3	−66	21
	2	64	Inferior Frontal Gyrus	0.65	−48	27	3
CCT scores							
VN	1	96	Lingual_L	0.67	−15	−48	−6
	2	91	Calcarine_R	0.62	27	−66	6
UPDRS part III scores							
SMN	1	91	Temporal_Sup_L	−0.48	−57	−21	12
	2	50	Paracentral Lobule	− 0.48	−6	−21	48

T value means the strength of correlation between intrinsic activities in areas of one RNS and clinical and cognitive scores. Keys, *BVMT-R* brief visuospatial memory test-revised; *CCT* clock copying test; *DMN* default mode network; *L* left; *R* right; *SMN* sensorimotor network; *Sup* superior; *UPDRS* the unified Parkinson’s disease rating scale; *VN* visual network

## Discussion

Using ICA, sources of signal variation were blindly separated and 7 large-scale brain networks were extracted. The DMN, VN and SMN were selectively vulnerable in newly diagnosed drug-naïve PD patients with MCI. Moreover, FC between the SMN and limbic network, and between the VAN and VN were more prone to be damaged in all PD patients.

The previous study has shown that DMN was characterized by reduced activation in task-based situation compared with the resting state [14]. In PD, DMN is the most studied intrinsic connectivity network, which is consistent with previous research on other neurological and psychiatric disorders [9]. The first study to explore the relationship between resting state FC in DMN and cognitive performance in PD was focused on cognitively unimpaired PD patients, and suggested reduced FC in the right medial temporal lobe and bilateral inferior parietal cortex within the DMN using the ICA [31]. Our previous study using the same method found lower FC in the left inferior parietal lobule within the DMN in cognitively unimpaired drug-naïve PD patients with akinetic-rigidity subtype [32]. Even though these patients did not meet the criteria for cognitive impairment, the altered FC within the DMN was significantly associated with cognitive function. In addition, more recent studies indicated connectivity changes in several RSNs rather than a single intrinsic connectivity network. However, changes of intrinsic connectivity network and even the association between altered networks and specific cognitive performance held a considerable heterogeneity, which was likely due to the variability in the inclusion of patients, appliance of cognitive tests, and preprocessing strategies.

A dynamic functional analysis in PD suggested two discrete connectivity states, the within-network state (State I) with more frequent and sparse connectivity, and the between-network state (State II) with less frequent and strong interconnectivity [33]. In the State I, sparse connections were located mainly within DMN, VN, and SMN, which might play a vital role in the pathogenesis of PD manifestations. Our study observed abnormal FC within the DMN correlated with deficits in memory, within the VN correlated with deficits in visuospatial function, as well as within the SMN correlated with the severity of disease. Cortical visual processing regions have been involved in a number of neuroimaging studies about PD [34], reporting occipital-cortical thinning, metabolic deficits and hypoperfusion. A longitudinal fMRI study demonstrated a progressive loss of FC mainly in posterior parts of brain strongly correlated with decreased cognitive performance [35]. The visuospatial deficit in PD was regarded as primary posterior cortical pathology rather than dopamine deficits, which might be a sensitive predictor of progression to dementia in PD [36]. Several lines of evidence suggested disrupted sensorimotor integration in PD [37]. The cortico-striatal loops were commonly impaired in previous studies investigating SMN connectivity in PD patients. It can be noted that dopamine deficiency might be one of the potential mechanisms underlying impaired SMN in PD.

According to the hypothesis of Braak staging [38], the pathological process of PD occurs primarily in the brain stem, pursues an ascending process, and arrives to the neocortex in the final stage. One previous study reported reduced FC in mesolimbic-striatal and cortico-striatal circuits in drug-naïve PD patients, which reflected pathologic changes of early non-motor and motor deficits and corresponded to Braak staging [39]. The structural and functional connectivity in the SMN and limbic network have been investigated separately in PD, and the correlation between these two RSNs was less known. A connectomic analysis revealed that amygdala, as a key structure in the limbic system, had a close interplay with areas within the SMN, including the postcentral gyrus, precentral gyrus and paracentral lobule, suggesting a limbic–motor interface involved in the emotional modulation of complex functions [40]. The impaired emotion perception can contribute to the damage of smiling mimicry in PD, which is closely related to fibers connection between the amygdala and SMN. Our finding of decreased FC between the SMN and limbic network was in line with previous literature and fostered the finding of existed alterations of limbic-motor interface in newly diagnosed drug-naïve PD patients.

More than a decade ago, two attention systems (DAN and VAN) with distinct anatomy and function has been introduced, and their roles in the visuospatial attention system were mainly described [41]. The DAN can be active when attention is overtly or covertly oriented in space, and the VAN can be active when faced with unexpected stimuli associated with behavior. In advanced PD, the visual processing impairment (e.g., misperception and hallucinations) was closely related to the DMN, DAN, VAN and VN [42]. When the DAN was unable to recruit activation, the DMN and VAN can frequently interactive and relatively overactive. Our finding of decreased FC between the VAN and VN might be an early change in the visuospatial attention system of PD, but subsequent changes and significance remain to be further studied.

There are some limitations within the present study. First, the level of education differs between the PD-MCI and PD-CU subgroups, which might be an important confounder. In the analyses, this variable was regarded as covariate to reduce the influence. Another concern is the modest sample size, which limited the generalizability of our findings. In addition, it should be noted that PD patients in our previous published study focused on the DMN [11] were also included in the current study, and HCs in the current study were not evaluated by HARS, HDRS and MoCA.

## Conclusion

We found that functional changes in the DMN, VN and SMN were more evident in the newly diagnosed drug-naïve PD-MCI group. These alterations were associated with memory, visual functions and motor symptoms, and functional deficits in the DMN and VN might be potential biomarkers for predicting PDD. Altered FC between the SMN and limbic network, and between the VAN and VN reflected early dysfunction in the disease process of PD, which can offer additional insight into the pathophysiological alterations of brain connectivity in PD.

## Supplementary Information

**Additional file 1.**

**Additional file 2.**

## Data Availability

All data generated or analyzed during this study are available from the corresponding author by reasonable request.

## Abbreviations

ANOVA: Analysis of variance
BLO: Benton line orientation
BNT: Boston naming test
BOLD: Blood-oxygen-level-dependent
BVMT-R: Brief visuospatial memory test-revised
CCT: Clock copying test
CDT: Clock drawing test
CSF: Cerebrospinal fluid
DAN: Dorsal attention network
DMN: Default mode network
DOT-A: Adaptive digit ordering test
DST: Backward digit span test
EPI: Echo-planar-imaging
FC: Functional connectivity
FPN: Fronto-parietal network
FWHM: Full-width half-maximum
gICA: Group ICA
GRF: Gaussian random filed
HARS: Hamilton anxiety rating scale
HCs: Healthy controls
HDRS: Hamilton depression rating scale
HVLT-R: Hopkins verbal learning test-revised
H&Y: Hoehn & Yahr stage
IC: Independent components
ICA: Independent component analysis
MCI: mild cognitive impairment
MNI: Montreal neurological institute
MRI: Magnetic resonance imaging
NMS: Non-motor symptoms
PD: Parkinson’s disease
PDD: PD with dementia
PET,: Positron emission tomography
rs-fMRI: Resting-state functional magnetic resonance imaging
RSNs: Resting state networks
SMN: Sensorimotor network
UPDRS: Unified PD rating scale
VAN: Ventral attention network
VFT: Verbal fluency test
VN: Visual network
WAIS-RC: Wechsler intelligence scale for adult-Chinese revised.
