# Supplementary material for: Different resting-state network disruptions in newly diagnosed drug-naïve Parkinson’s disease patients with mild cognitive impairment
Source: BMC Neurol. 2021 Aug 25;21:327. doi: 10.1186/s12883-021-02360-z (PMC8386092; doi:10.1186/s12883-021-02360-z)
Supplement: Supplementary file 1 — Additional file 1. [file 12883_2021_2360_MOESM1_ESM.docx]

**Different resting-state network disruptions in newly diagnosed drug-naïve Parkinson’s disease patients with mild cognitive impairment**

Yanbing Hou^1^, Qianqian Wei^1^, Ruwei Ou^1^, Lingyu Zhang^1^, Xiaoqin Yuan^1^, Qiyong Gong^2*^, Huifang Shang^1*^

1 Department of neurology, Laboratory of Neurodegenerative Disorders, National Clinical Research Center for Geriatrics, West China Hospital, Sichuan University, Chengdu, Sichuan, China

2 Huaxi MR Research Center (HMRRC), Department of Radiology, West China Hospital, Sichuan University, Chengdu, Sichuan, China

*Correspondence to Huifang Shang, MD, Department of Neurology, National Clinical Research Center for Geriatrics, West China Hospital, Sichuan University, Chengdu 610041, Sichuan, China. Tel: 0086-18980602127, Fax: 0086-028-85423550. E-mail: hfshang2002@126.com. Qiyong Gong, PhD, Huaxi MR Research Center (HMRRC), Department of Radiology, West China Hospital, Sichuan University, Chengdu 610041, Sichuan, China. E-mail: huaxigongqy@163.com

**Supplementary methods**

**Participants**

The subjects were assessed face-to-face by a trained neuropsychologist in a quiet and comfortable room taking about 1 hour. All subjects were evaluated in the same order (Hopkins verbal learning test-revised (HVLT-R), clock drawing test (CDT), clock copying test (CCT), backward digit span test (DST), adaptive digit ordering test (DOT-A), Benton line orientation (BLO), brief visuospatial memory test-revised (BVMT-R), verbal fluency test (VFT), Similarity test in Wechsler intelligence scale for adult-Chinese revised (WAIS-RC), Boston naming test (BNT)).

**MRI data acquisition**

The sequences of conventional MRI were performed as follows: (1) The T1-weighted imaging: repetition time/echo time (TR/TE) = 1600/9.2 m, flip angle (FA) = 130°, field of view (FOV) = 250 × 250 mm^2^, matrix size = 148 × 320, voxel size = 1.2 × 0.8 × 5.0 mm^3^, axial slices = 21; (2) The T2-weighted imaging: TR/TE = 4000/93 m, FA = 120°, FOV = 220 × 220 mm^2^, matrix size = 230 × 320, voxel size = 0.8 × 0.7 × 5.0 mm^3^, axial slices = 21; (3) The fluid-attenuated inversion recovery imaging: TR/TE = 6000/93 m, FA = 130°; FOV = 220 × 220 mm^2^, matrix size = 198 × 256, voxel size = 1.0 × 0.9 × 5.0 mm^3^, axial slices = 21.
