# Supplementary material for: Different resting-state network disruptions in newly diagnosed drug-naïve Parkinson’s disease patients with mild cognitive impairment
Source: BMC Neurol. 2021 Aug 25;21:327. doi: 10.1186/s12883-021-02360-z (PMC8386092; doi:10.1186/s12883-021-02360-z)
Supplement: Supplementary file 2 — Additional file 2. [file 12883_2021_2360_MOESM2_ESM.docx]

**Different resting-state network disruptions in newly diagnosed drug-naïve Parkinson’s disease patients with mild cognitive impairment**

Yanbing Hou^1^, Qianqian Wei^1^, Ruwei Ou^1^, Lingyu Zhang^1^, Xiaoqin Yuan^1^, Qiyong Gong^2*^, Huifang Shang^1*^

1 Department of neurology, Laboratory of Neurodegenerative Disorders, National Clinical Research Center for Geriatrics, West China Hospital, Sichuan University, Chengdu, Sichuan, China

2 Huaxi MR Research Center (HMRRC), Department of Radiology, West China Hospital, Sichuan University, Chengdu, Sichuan, China

*Correspondence to Huifang Shang, MD, Department of Neurology, National Clinical Research Center for Geriatrics, West China Hospital, Sichuan University, Chengdu 610041, Sichuan, China. Tel: 0086-18980602127, Fax: 0086-028-85423550. E-mail: hfshang2002@126.com. Qiyong Gong, PhD, Huaxi MR Research Center (HMRRC), Department of Radiology, West China Hospital, Sichuan University, Chengdu 610041, Sichuan, China. E-mail: huaxigongqy@163.com

Supplementary Table S1 Neuropsychological performance results for healthy controls and Parkinson’s disease patients

| Z score | Controls | | PD-CU | | PD-MCI | | P value^1^ | P value^2^ |
| --- | --- | --- | --- | --- | --- | --- | --- | --- |
|  | ‾X | SD | ‾X | SD | ‾X | SD |  |  |
| DOT-A | 0.000 | 1.000 | 0.584 | 1.156 | -0.817 | 1.082 | <0.001* | <0.001* |
| DST | 0.000 | 1.000 | 0.108 | 0.886 | -0.432 | 0.893 | 0.102 | 0.047* |
| VFT | 0.000 | 1.000 | 0.383 | 0.839 | -0.519 | 0.911 | 0.006* | 0.001* |
| CDT | 0.000 | 1.000 | 0.498 | 0.882 | -0.498 | 1.133 | 0.006* | 0.002* |
| HVLT-R total | 0.000 | 1.000 | 0.348 | 0.813 | -0.942 | 0.782 | <0.001* | <0.001* |
| BVMT-R | 0.000 | 1.000 | -0.029 | 0.793 | -1.131 | 1.254 | <0.001* | 0.001* |
| WAIS-RC | 0.000 | 1.000 | 0.739 | 0.653 | -0.549 | 1.077 | <0.001* | <0.001* |
| BNT | 0.000 | 1.000 | 0.482 | 0.797 | -0.417 | 0.827 | 0.005* | 0.001* |
| BLO | 0.000 | 1.000 | 0.267 | 0.667 | -1.376 | 1.444 | <0.001* | <0.001* |
| CCT | 0.000 | 1.000 | 0.289 | 0.748 | -0.671 | 1.410 | 0.012* | 0.009* |

* indicate significant difference

^1^ Comparison among PD-MCI, PD-CU patients, and control subjects

^2^ Comparison between PD-CU and PD-MCI patients

Keys: DOT-A, adaptive digit ordering test; DST, backward digit span test; VFT, verbal fluency test; CDT, clock drawing test; HVLT-R, the Hopkins verbal learning test-revised; BVMT-R, the brief visuospatial memory test revised; WAIS-RC, Wechsler intelligence scale for adult-Chinese revised; BNT, Boston naming test; BLO, Benton Line Orientation; CCT, clock copying test.
